# Estimating the Intended Sound Direction of the User: Toward an Auditory Brain-Computer Interface Using Out-of-Head Sound Localization

**DOI:** 10.1371/journal.pone.0057174

**Published:** 2013-02-20

**Authors:** Isao Nambu, Masashi Ebisawa, Masumi Kogure, Shohei Yano, Haruhide Hokari, Yasuhiro Wada

**Affiliations:** 1 Nagaoka University of Technology, Nagaoka, Niigata, Japan; 2 Nagaoka National College of Technology, Nagaoka, Niigata, Japan; McGill University, Canada

## Abstract

The auditory Brain-Computer Interface (BCI) using electroencephalograms (EEG) is a subject of intensive study. As a cue, auditory BCIs can deal with many of the characteristics of stimuli such as tone, pitch, and voices. Spatial information on auditory stimuli also provides useful information for a BCI. However, in a portable system, virtual auditory stimuli have to be presented spatially through earphones or headphones, instead of loudspeakers. We investigated the possibility of an auditory BCI using the out-of-head sound localization technique, which enables us to present virtual auditory stimuli to users from any direction, through earphones. The feasibility of a BCI using this technique was evaluated in an EEG oddball experiment and offline analysis. A virtual auditory stimulus was presented to the subject from one of six directions. Using a support vector machine, we were able to classify whether the subject attended the direction of a presented stimulus from EEG signals. The mean accuracy across subjects was 70.0% in the single-trial classification. When we used trial-averaged EEG signals as inputs to the classifier, the mean accuracy across seven subjects reached 89.5% (for 10-trial averaging). Further analysis showed that the P300 event-related potential responses from 200 to 500 ms in central and posterior regions of the brain contributed to the classification. In comparison with the results obtained from a loudspeaker experiment, we confirmed that stimulus presentation by out-of-head sound localization achieved similar event-related potential responses and classification performances. These results suggest that out-of-head sound localization enables us to provide a high-performance and loudspeaker-less portable BCI system.

## Introduction

The Brain-Computer Interface (BCI) is an advanced technology used for controlling external devices from the measured brain activity of a user, without any muscular action [1]. It is thus particularly attractive for people with a severe movement disorder such as amyotrophic lateral sclerosis (ALS) or spinal cord injury. The BCI is expected to not only replace lost functions but also restore or improve natural actions.

In a non-invasive BCI, electroencephalography (EEG) has been commonly used because of its high temporal resolution and portability. BCI studies using EEG have exploited several types of EEG signals: sensory motor rhythm, slow cortical potentials, steady-state responses, and event-related potentials (ERPs). Among them, an ERP called P300 is thought to be especially useful for a BCI because it is robustly evoked by various types of stimuli (including visual and auditory) with a latency of approximately 300 ms (250 to 500 ms) when a person pays attention to the stimuli in the oddball paradigm [2], [3], [4]. One of the most successful examples of the BCI is the P300 visual speller system [3], [5], which presents a visual stimulus, such as a flashing character, to the user, and detects the P300 responses to identify which stimulus the user is perceiving. It has also been shown that ALS patients can handle this system [3], [5].

However, the visual P300 BCI cannot be used for people whose visual function is impaired. Recent studies have shown that the performance of the visual P300-based BCI depends on the ability to control eye movement or gaze [6], [7], [8]. Consequently, different types of BCIs are suitable for those who cannot use a visual BCI. For this purpose, gaze-independent (auditory, tactile, gaze-independent visual) BCI systems have been explored [8]. In particular, auditory-evoked P300 BCIs have been intensively examined [9], [10], [11], [12], [13], [14], [15], [16], [17], [18], [19]. In an auditory BCI, several characteristics of auditory stimuli, such as pitch, amplitude of tone, and voices, can be applied as a cue [10], [13], [16]. The direction of sound is also a unique characteristic of auditory stimuli. Since we can easily recognize where sound comes from in daily life, using the spatial information of sound seems to be a promising approach for an auditory BCI. Using auditory stimuli from different directions, previous studies succeeded in estimating the direction/location of sound from either left, right, or both sides [13], [17], [19]. Furthermore, it can be extensible to more than five directions [11], [12] by presenting auditory stimuli from several directions using loudspeakers around the subject. In these studies, Schreuder et al. first proposed a new auditory BCI paradigm called “spatial hearing” or “Auditory Multi-class Spatial ERP (AMUSE)” [11], [12]. In this experimental paradigm, spatially distinct sound was presented from five or six loudspeakers, and the user intuitively perceived the sound without any special training. Using the spatial information of sound, it has shown the potential to become a multi-class high-performance auditory BCI. However, generating auditory stimuli from the loudspeakers will be problematic for a portable BCI system because this requires a large space, and equipment with many loudspeakers. Therefore, to use precise spatial information of sound for a BCI, it is necessary to synthesize auditory stimuli outside the head through earphones or headphones.

One possible solution to this problem is the out-of-head sound localization technique [20], [21], [22], in which auditory stimuli are presented through earphones so that stimulation of the eardrum becomes similar to the way in which actual sound is presented. Using this technique, it is possible to present virtual auditory stimuli from any direction, including from the rear, without loudspeakers.

In this study we used out-of-head sound localization and assessed its usefulness for an auditory BCI. In the experiment using out-of-head sound localization, the user (subject) directed his or her attention to one of the six sound sources. The sound was presented binaurally through earphones. EEG signals were recorded throughout the experiment and were used to estimate whether the user attended the direction of the presented stimulus using a support vector machine (SVM). To feasibility of the BCI using out-of-head sound localization, we additionally conducted an experiment using loudspeakers for stimulus presentation and compared the results with those obtained from the out-of-head sound localization experiment. Through this investigation, we demonstrated that out-of-head sound localization can be incorporated in an auditory BCI system.

## Materials and Methods

### Out-of-head sound localization experiment

#### Subjects

Seven healthy people (6 males and 1 female, ages 22–24) participated in this study, which was approved by the ethics committee of the Nagaoka University of Technology. All the subjects were given information on the experiment and signed consent forms. They had normal hearing and no history of hearing problems.

#### EEG recording

We measured the EEG signals using a digital electroencephalograph system (ActiveTwo, Biosemi, Amsterdam, The Netherlands) with 64 electrodes attached to the subject's scalp using a cap. The electrodes were placed in accordance with the international 10–20 system. Reference electrodes were attached to each earlobe. The electrode on the right earlobe had an amplifier in it, i.e., active electrodes and formed a feedback loop with a passive electrode on the left earlobe. Using these two electrodes, reference voltage was calculated (for details, see http://www.biosemi.com/). The sampling frequency was 256 Hz.

#### Out-of-head sound localization

The principle of out-of-head sound localization is to reproduce the sound waveforms of an actual sound field at the listener's eardrums using stereo earphones or headphones. The procedure for out-of-sound localization follows: 1) measure the impulse responses of the Spatial Sound Transfer Functions (SSTFs) and Ear Canal Transfer Functions (ECTFs), 2) obtain the Sound Localization Transfer Functions (SLTFs), and 3) produce the out-of-head sound using the SLTFs. To achieve this, it is necessary to obtain the transfer functions from loudspeakers to the eardrums. Since the shapes of the head and external auditory canal are different between subjects, the transfer function fitted to each subject is not identical [21]. Using an unfitted transfer function causes the problem that the spatial location of sound is not localized at actual sound sources. Thus we require the transfer function specific to each subject to accurately present the out-of-head sound images. Therefore, we measured the transfer function specific to each subject using the impulse response measurement method before the experiment. Details of the measurements for the transfer function are described in Text S1 and Figure S1.

In the experiment, white noise (100 Hz–15 kHz) was adopted as an auditory stimulus (cue). We produced the auditory stimulus for each direction by convolving the white noise with the impulse response of the out-of-head transfer function (see Text S1 for details). The sound pressure level was adjusted to 65 dB using a dummy-head (SAMRAI, KOKEN, Tokyo, Japan). The subject wore intra-concha earphones (MDR-ED238, Sony, Tokyo, Japan), and the auditory stimuli were amplified through a USB audio interface (QUAD-CAPTURE UA-55, Roland, Hamamatsu, Japan).

#### Experimental protocol

**Figure 1:**
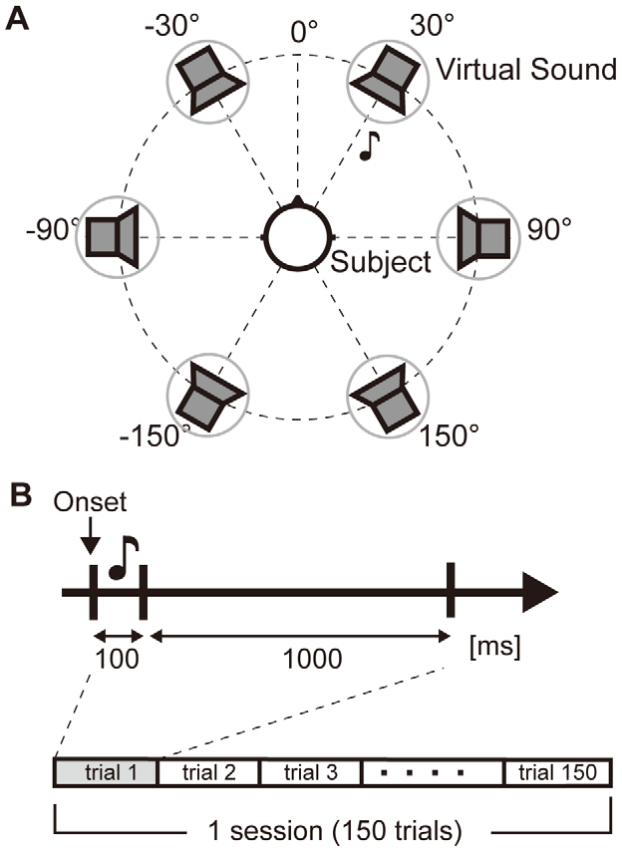
Experimental setting and protocol. (A) Six directions for the virtual auditory stimuli. The subject looked forward (0°). (B) The trial consisted of stimulus and inter-stimulus interval. An auditory stimulus (cue) was presented during the 100 ms after stimulus onset. Inter-stimulus interval was 1000 ms. 150 trials per session were performed.

The protocol that assesses the performance of our system is shown in Figure 1. Each trial consisted of a 100 ms stimulus and a 1000 ms inter-stimulus interval (rest period). The sound sources were allocated to six directions: 30°, 90°, 150°, −150°, −90°, and −30°. In each trial, an auditory stimulus (cue) from one of the six directions was presented. The presented auditory cues were in a random sequence while the subject focused on one of the directions (target direction). The stimulus from the target direction was presented for about 20% of the stimuli. The target direction was fixed during the session, and the subject was informed of the target direction before the session. The subject performed a counting task during the trial, i.e., counted the number of times this target cue was presented. To avoid the effects of visual sensation and eye blink, the subject was instructed to perform the counting task with closed eyes during the trial. Each subject completed 12 sessions in total (in 2 days at 6 sessions per day). Each session consisted of 150 trials.

#### Localization test

To check whether the subject heard the out-of-head sound image accurately, a localization test was performed before and after the main experiment. In the test, an auditory stimulus was presented from one of the six directions and the subject reported from which direction he perceived the auditory stimulus. This was repeated 10 times in each direction. In total 120 responses were obtained.

#### Data analysis (preprocessing and calculating ERP)

Raw EEG data sampled at 256 Hz were filtered using a Butterworth band-pass filter (third-order, 1 to 7 Hz). After the filtering, the average for −100 ms to 0 ms in each trial was set as the baseline, and the baseline amplitude was subtracted from the data in each trial. To remove artifacts unrelated to brain activity, we excluded trials whose maximum absolute value of amplitude exceeded 60 µV, which may have included significant noises or artifacts. Data that were filtered and excluded artifact trials were used in the analysis.

To confirm evoked potentials when the subject attended the direction of the presented stimuli, we calculated the averaged event-related potentials (ERPs) for each electrode in each subject. The onset of the EEG signals was set to the timing of the auditory cue (0 ms). ERPs were evaluated for each of the target and non-target trials and the positive peak latencies and their amplitudes for the averaged ERP were investigated as characteristics of the ERP. A Mann-Whitney U test was performed to check the differences between target and non-target waveforms in each time interval (*p*<0.01). To further investigate differences between the target directions, we also calculated the averaged ERP signals for each direction. This analysis was done by calculating the averaged ERP for target and non-target trials within combined sessions for which the target direction was the same because the target direction was fixed in a session. Two-way repeated-measures analysis of variance (ANOVA) was conducted for latencies and amplitudes (factors: direction and trial type).

#### Classification of target/non-target direction

To predict target or non-target trials, i.e., whether the subject attended the direction from which the auditory cue was presented, a linear support vector machine (SVM) [23], [24] was used. Given a set of EEG signals ***x*** and a corresponding label *y*, a classifier determined the optimal boundary of each data class.

The EEG signals ***x*** were prepared as follows. First, we extracted the data for 1100 ms from the beginning of the auditory cue presentation for each trial (0–1100 ms). The first 280 samples for each electrode were reduced to 28 samples by averaging every 10 samples. Thus, the dimension of the feature vector ***x*** became 1792 (28×64). Next, we normalized the data so that the maximum absolute value for each electrode became 1, which made for the amplitude of each electrode constant. A label *y* is set to 1 for target-trials and −1 for non-target trials.

Using weight parameters *a* and *b*, the objective function of the SVM is described as follows:
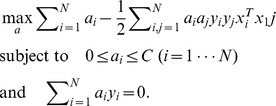

*N* is the number of samples (trials). Subscript *i* represent data for *i*-th trial. This is a soft-margin SVM and requires the setting of a penalty parameter *C*. To determine parameters *a*, *b*, and *C*, the data were separated into three sets of samples: training set, *C*-searching set, and test set. The SVM classifier was constructed (determination of *a* and *b*) using the training set. Half the target trials and the same number of non-target trials were used as the training set. The remaining data were equally divided into the *C*-searching and test set. The classification accuracy for the *C*-searching set was evaluated for each candidate *C*, and the value of *C* that obtained the highest accuracy was used as its optimal value. *C* was searched between 2^−16^ to 2^2^ (2^−16^≤2*^m^*≤2^2^, *m* = −16∶1∶2). Finally, the test accuracy was evaluated using the test set.

The target classification accuracy was defined as the number of correctly classified target samples divided by the total number of target samples, and the non-target classification accuracy was the number of correctly classified non-target samples divided by the total number of non-target trials. Hereafter, accuracy for all the samples, i.e., the number of correctly classified samples divided by the total number of samples, was referred as the “classification accuracy” for simplicity. To suppress variability in the evaluation, we repeated the analysis 10 times with randomly exchanged training, *C*-searching, and test samples. The mean accuracy of these 10 analyses was taken as the result for each subject.

Since classification accuracy seems to be improved with increasing signal-to-noise ratio of the EEG signals by trial-averaging as in the P300 speller [5], we calculated the trial-averaged EEG signals for 2 to 10 samples. Using a bootstrap approach, samples were randomly selected and averaged to create averaged EEG signals. This bootstrap was repeated so that the number of averaged samples became the same as for the single-trial classification. Note that, for non-target trials, trials for different auditory directions were averaged in the bootstrap approach because of the small number of samples. The averaged signals were used as inputs for classification, and accuracies were evaluated for each averaging (iteration) procedure. Statistical significance was tested by the two-way repeated-measures ANOVA (factors: trial type i.e., target/non-target, and number of averaging).

To examine whether estimating the intended specific target direction is better than for the other target directions in terms of classification accuracy, across-subjects accuracies were calculated for each direction. A statistical test was performed using the two-way repeated-measures ANOVA (factors: direction and number of averaging). In addition, to reveal a relationship between classification accuracy and behavioral data for each direction, correlation coefficients between the classification accuracy and behavioral data (localization accuracy and counting errors) were evaluated across subjects and directions. This correlation analysis was performed for data from each number of averaged-trials but only the result for single- and 10-trial averaged trials are shown because the same results were observed for any number of averaged-trials.

#### Spatial and temporal feature selection

**Figure 2:**
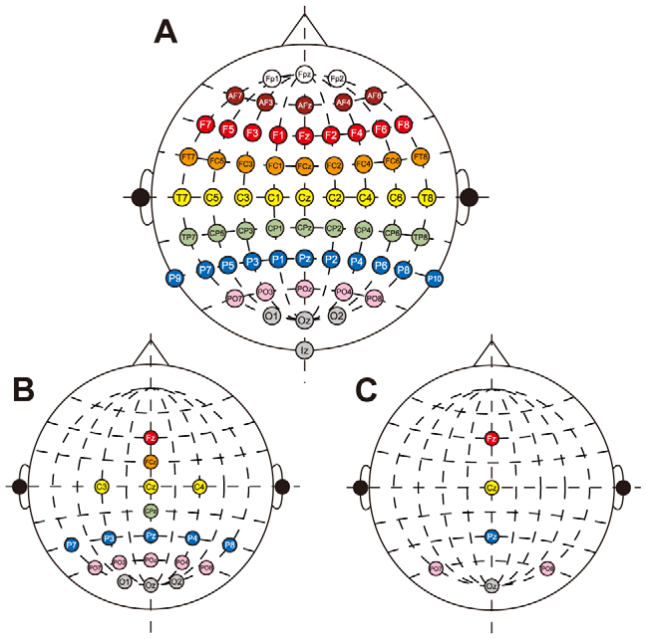
EEG electrode locations. Spatial location of 64 channels EEG (A), 19 channels (B), and 6 channels (C). Reference electrodes attached to the ears.

When we used all the EEG data in a trial (64 channels and 1100 ms duration), it was unclear what type of information the classifier referred to for prediction. If classification was achieved using the P300 responses as we expected, a part of the available EEG features should be crucial to the classification. If so, the classification accuracy would remain high even when we used only a few representative channels. To examine this, we prepared different feature sets and applied them to SVM classification. First, we calculated classification accuracy when the channels for classification were reduced. Based on a previous literature, datasets with a 6 channel-set and a 19 channel-set [25] were used (Figure 2). In the 6 channel-set, Fz, Cz, Pz, PO7, PO8, and Oz were selected to cover the central and posterior part of the electrodes (Figure 2C). The 19 channel-set included the 6 channel-set and the additional 13 channels: FCz, CPz, PO2, C3, C4, P3, P4, P7, P8, PO3, PO4, O1, and O2 (Figure 2B). When we used these selected channel-sets, all temporal features from 0 to 1100 ms were used.

For temporal feature selection, we prepared six datasets, which have 500 ms samples of the 64 channels: 0–500, 100–600, 200–700, 300–800, 400–900, 500–1000, and 600–1100 ms datasets. The number of temporal features (samples) for each dataset was the same, but the 100–600 ms data set was expected to have a high accuracy since P300 is generally observed during this period.

### Loudspeaker experiment

#### Experimental setting

Six out of seven subjects (Sub1, 2, 3, 4, 6, and 7) in the main experiment participated in the loudspeaker experiment. The Ethics Committee of the Nagaoka University of Technology approved the experiment. All the subjects were given information on the experiment and signed an informed consent, as in the main experiment.

EEG recording, experimental protocol, and data analysis (artifact rejection, calculating ERPs and classification accuracy) were identical to those in the main experiment except for Sub3. For Sub3, EEG data were sampled at 2048 Hz, and then down-sampled at 256 Hz. The only difference for the experimental paradigm was that the auditory stimulus was generated by loudspeakers. The reverberation time in the test room was about 0.1 s. Twenty-four loudspeakers (MODEL SD-0.6, EMIC, Japan) were placed at 15° intervals, and six of them (30°, 90°, 150°, −150°, −90°, and −30°) were used. The distance from the center of subject's head to the face of the each loudspeaker was 1.5 m. The subject sat on a seat and pseudo white noise was radiated from the loudspeakers through an amplifier (SRP-P4005, Sony, Tokyo, Japan). The sound pressure level was adjusted to 65 dB.

## Results

### Localization test

**Figure 3:**
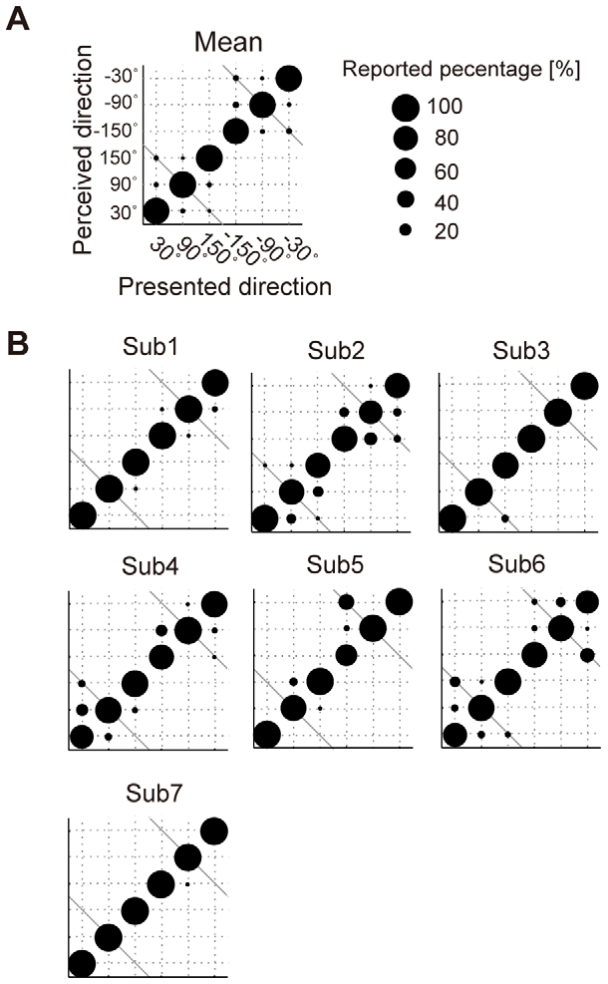
Accuracy in localization test. (A) Localization accuracy across subjects for each direction. Each circle represents the percentage the subjects reported accurately as the perceived direction. The horizontal axis represents the direction of presented auditory stimulus and the vertical axis denotes the direction the subjects perceived. Two diagonal gray lines at lower left and upper right indicate the direction of front-back confusion. (B) Localization accuracy for each subject.

Before the experiment, a localization test was conducted to confirm that the presented out-of-head sound image was correctly localized. Table 1 (left) shows the results of the localization test. The number of error trials in which the subject reported a wrong direction ranged from 3 to 36, and correct perceptions were 85.0 to 98.8% (mean ± SD across subjects: 92.4±6.4%). This suggests that sound was correctly perceived from six directions. Figure 3A shows localization accuracy across subjects for each presented sound direction. The directions they reported wrongly were located in neighboring directions. Some trials were perceived from the direction of the same side with a 120° difference, known as front-back confusion. When we checked the localization accuracies for each subject (Figure 3B), individual differences of sound localization were observed.

**Table 1:** Behavioral result in localization test.

	Out-of-head sound localization			Loudspeaker		
Subject	Number of error trials	Error rate [%]	Correct rate [%]	Number of error trials	Error rate [%]	Correct rate [%]
Sub1	5	2.1	97.9	0	0.0	100.0
Sub2	36	15.0	85.0	0	0.0	100.0
Sub3	3	1.3	98.8	1	0.4	99.6
Sub4	27	11.3	88.8	2	0.8	99.2
Sub5	20	8.3	91.7			
Sub6	36	15.0	85.0	1	0.4	99.6
Sub7	1	0.4	99.6	1	0.4	99.6
**Average**	**18.3**	**7.6**	**92.4**	**0.8**	**0.3**	**99.7**
SD	14.6	6.4	6.4	0.8	0.3	0.3

### Counting target trials

After each session of the experiment, the subjects reported on how many trials the auditory stimulus was presented from the target direction. The counting error for each target direction is shown in Table 2 (upper). The error was the absolute value for the difference in counting and presented numbers divided by the presented number. On average, the error was 5.3% across all the directions. Although individual differences were found, a statistical test (one-way ANOVA) showed no difference between counting directions across subjects (F = 0.47, *p*<0.80). One of the subjects (Sub6) showed relatively large errors for all the directions.

**Table 2:** Error rate in counting number of presented targets.

Out-of-head sound localization							
	Direction [degree]						
Subject	30	90	150	−150	−90	−30	Average (subject) [%]
Sub1	0	1.6	0	1.6	3.2	0	**1.1**
Sub2	1.7	3.3	14.8	4.8	0	9.4	**5.7**
Sub3	0	0	0	3.2	1.7	4.7	**1.6**
Sub4	8.2	3.3	8.3	1.6	4.9	7.9	**5.7**
Sub5	5.0	12.9	0	1.6	1.6	3.2	**4.1**
Sub6	24.8	16.1	11.6	14.5	24.3	21.9	**18.9**
Sub7	0	0	0	0	0	1.6	**0.3**
**Average (direction)**	**5.7**	**5.3**	**5.0**	**3.9**	**5.1**	**6.9**	**5.3**

Loudspeaker							
	Direction [degree]						
Subject	30	90	150	−150	90	−30	Average (subject) [%]
Sub1	0	1.6	0	0	0	0	**0.3**
Sub2	0	0	0	0	3.3	1.6	**0.8**
Sub3	1.7	1.7	1.7	1.6	3.3	1.6	**1.9**
Sub4	0	0	0	4.8	0	0	**0.8**
Sub6	8.2	6.5	8.2	6.5	6.6	7.8	**7.3**
Sub7	1.6	3.2	1.7	0	0	0	**1.1**
**Average (direction)**	**1.9**	**2.2**	**1.9**	**2.2**	**2.2**	**1.8**	**2.0**

### Artifact rejection

Table 3 (left) shows the number of trials excluded after artifact rejection. On average, across the subjects, 3.4±2.7% of the trials were excluded. For Sub6, the rejection rate was relatively high. We found that the rejected channels for Sub6 were mainly located around frontal electrodes (Fpz, Fp1, Fp2, AF7, AF8). This may be caused by unavoidable eye blinks or muscle contractions even the subject closed their eyes.

**Table 3:** Result of artifact rejection.

	Out-of-head sound localization		Loudspeaker	
Subject	Number of excluded trials	Rejection rate [%]	Number of excluded trials	Rejection rate [%]
Sub1	67	3.7	20	1.1
Sub2	29	1.6	28	1.6
Sub3	6	0.3	48	2.7
Sub4	54	3.0	50	2.8
Sub5	70	3.9		
Sub6	158	8.8	48	2.7
Sub7	40	2.2	80	4.4
**Average**	**60.6**	**3.4**	**45.7**	**2.5**
SD	48.4	2.7	20.9	1.2

### Averaged ERPs

**Figure 4:**
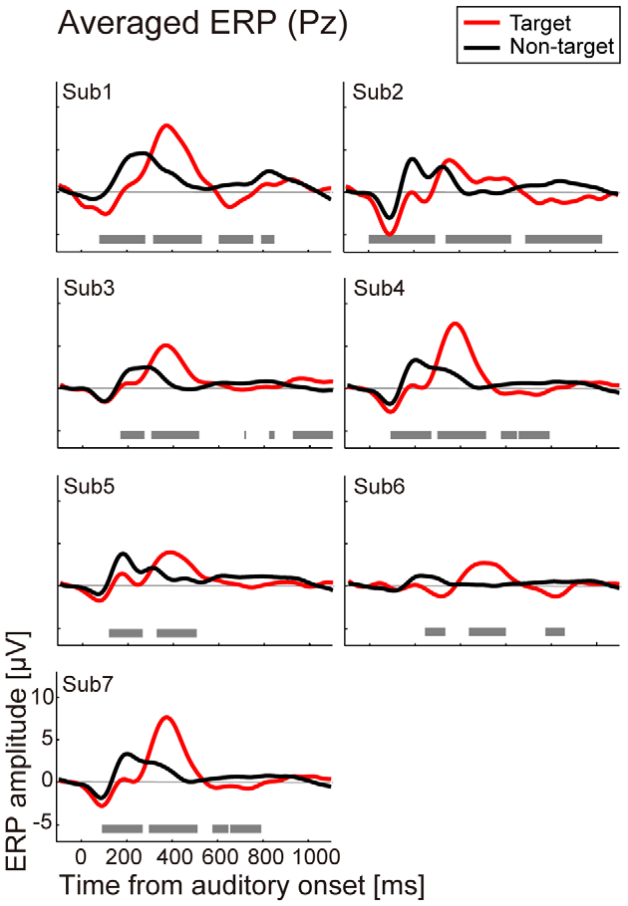
Averaged ERP at Pz for each subject. Each figure shows the averaged ERP responses at electrode Pz for each subject (Sub1–Sub7). The horizontal axis denotes time from auditory onset, and the vertical axis shows the ERP amplitude. The red line shows the ERP for target trials and the black line shows the ERP for non-target trials. The gray bar at the bottom of each figure indicates the significant difference between target and non-target waveforms (Mann-Whitney U test, *p*<0.01).

Figure 4 shows the trial-averaged EEG waveform for target and non-target trials in all the subjects measured at electrode Pz. In the target trial, prominent responses were observed. The peak amplitude of the ERP for the target trial was larger than that for the non-target trial (Table 4 upper). The latency of the peak amplitude was also different between the target and non-target trials, especially for 200 to 500 ms across subjects (Figure 4, gray bar in each figure; *p*<0.01). Latencies in the target trial (mean: 384 ms) were longer than those in the non-target trial (217 ms) for all the subjects. Although the observed positive responses in the target trial had a slightly longer latency than about 300 ms, this response was a typical waveform for the P300 [26]. These results indicate that the evoked potential, which is likely to be the P300, differed between the target and non-target trials even when we used the out-of-sound localization technique to present auditory stimuli.

**Table 4:** ERP latency and amplitude.

Out-of-head sound localization				
	Target		Non-target	
	Latency [ms]	Peak amplitude [µV]	Latency [ms]	Peak amplitude [µV]
Sub1	375	7.8	270	4.6
Sub2	356	3.8	192	3.9
Sub3	364	5.0	282	2.5
Sub4	375	7.6	200	3.3
Sub5	383	4.0	177	3.9
Sub6	488	2.6	219	1.1
Sub7	344	5.4	181	3.8
**Average**	**384**	**5.2**	**217**	**3.3**

Loudspeaker				
	Target		Non-target	
	Latency [ms]	Peak amplitude [µV]	Latency [ms]	Peak amplitude [µV]
Sub1	325	8.0	184	7.8
Sub2	340	6.0	196	5.2
Sub3	348	5.2	301	3.6
Sub4	356	7.5	196	4.0
Sub6	383	3.4	196	3.0
Sub7	360	8.0	177	4.2
Average	352	6.3	208	4.6

Thus, it may be possible to predict the intended sound direction of the subject when we extract and classify these EEG signals.

**Figure 5:**
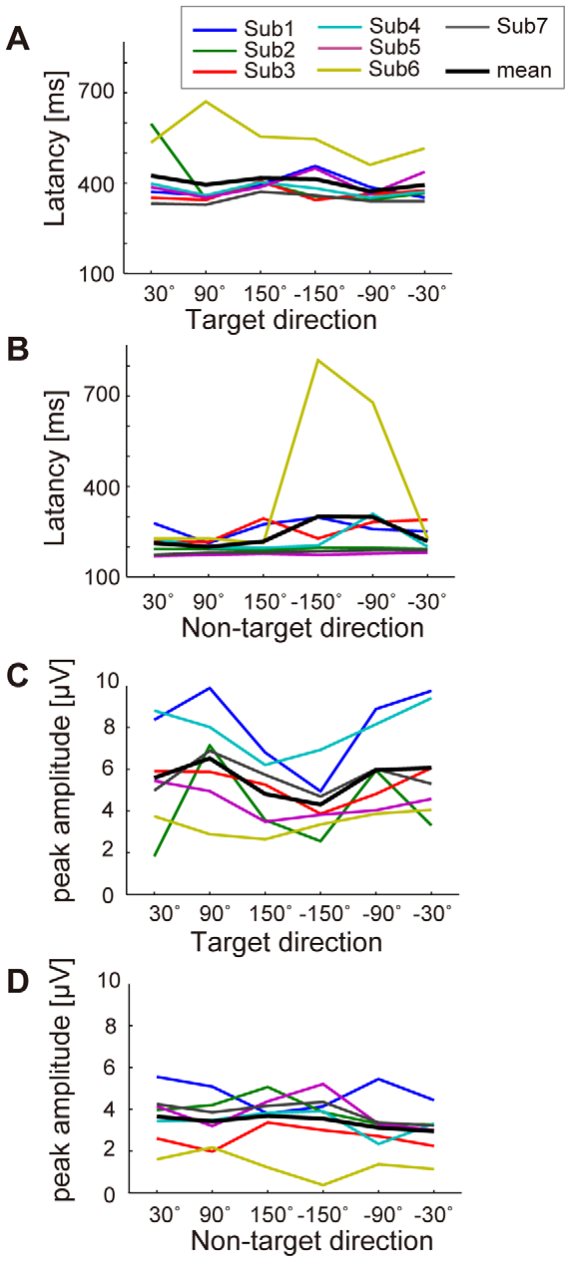
ERP latency and peak amplitude for each direction. (A) ERP latency of the positive peak for each direction in target trials. Each line indicates each subject. Data were obtained from electrode Pz. The horizontal axis denotes direction. The black line shows mean values across subjects in each direction. (B) ERP latency of the positive peak for each direction in non-target trials. (C) Peak amplitudes of ERP at Pz for each direction. Data were calculated from target-trials. (D) Peak amplitudes of ERP in non-target trials.

To examine whether differences between directions of sound were found, we looked at the latencies and amplitudes of positive peaks for each direction (Figure 5). The latencies across subjects for each direction ranged from 373 to 424 ms in the target trials (the bold line in Figure 5A) and 199 to 300 ms in non-target trials (the bold line in Figure 5B). Directional differences were small although large individual differences were observed (n.s., F = 0.9 and 1.3 for target and non-target trials). Results for the peak amplitudes in target trials decreased in backward directions while non-target amplitudes were similar (Figure 5C, D). A one-way repeated-measures ANOVA showed statistical significance only in the target peak amplitudes between directions (F = 4.4, *p*<0.01) and the differences for two pairs (90° and −150°, −150° and −30°) were significant in the multiple comparison procedure (Ryan's method, *p*<0.05). For Sub6 at −150°, the latency in the non-target trials was high and its amplitude was small because of flat waveforms, suggesting failure in detecting a normal ERP.

### Classification of intended sound direction

**Figure 6:**
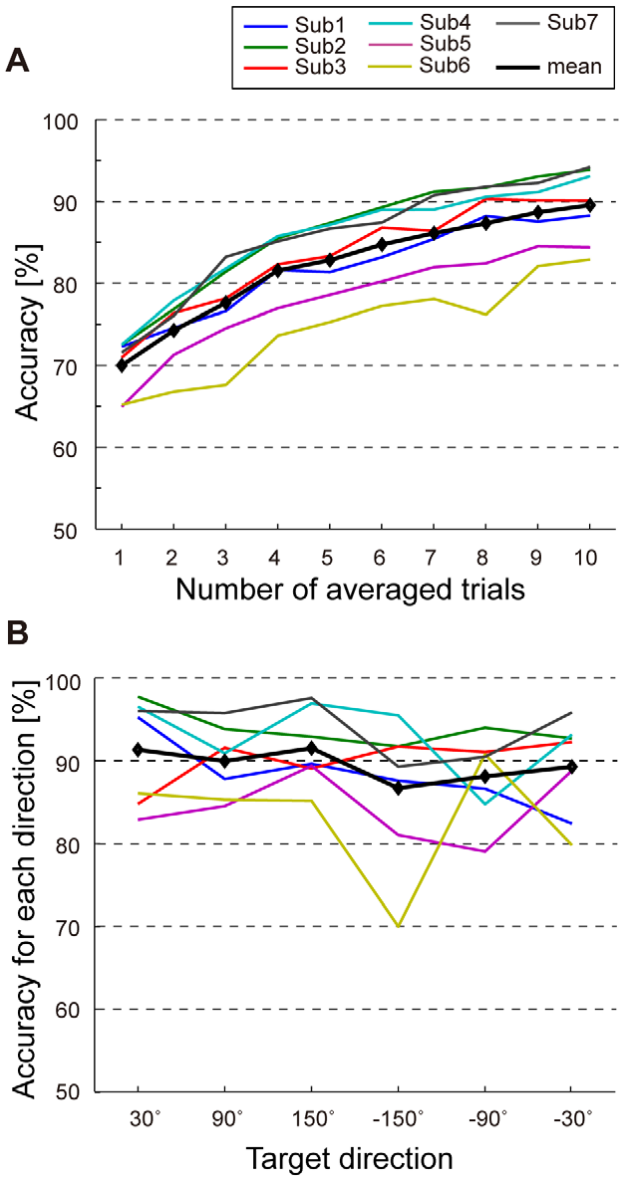
Accuracy in predicting the perceived direction and its directional biases. (A) Classification accuracy when the number of averaged trials was different. Each colored line represents the accuracy for an individual subject. The bold black line indicates the mean accuracy across subjects. (B) Classification accuracies for each direction. The data for 10-averaged trials is shown. Each line shows averaged accuracy across subjects for each direction and average for all the directions.

To predict target or non-target directions, we performed classification analysis using EEG signals from 64-channel electrodes. Figure 6A shows classification accuracy for each subject and averaged accuracy across subjects. In the single-trial classification (i.e., the number of averaging trials is 1) the accuracy was 70.0±3.4% (mean ± SD). When we used trial-averaged EEG signals as inputs to a classifier, the accuracy gradually increased with the number used in averaging. In 6-trial averaging, the accuracy was 84.7±4.6%, and in 10-trial averaging it reached 89.5±4.6%. This indicates that averaging EEG signals improved accuracies (F = 197.1, *p*<0.001). The improvement in 10-trial averaging was over 19.6% compared with when data from a single-trial were used. Across-subject accuracies for target and non-target trials are listed in Table 5 (left). The accuracies between target and non-target trials seemed to be comparable (for target: 67.5%; non-target: 70.2%) but the target accuracy became slightly higher than the non-target accuracy along with an increasing number of averaging (for 10-trial averaged classification, target: 89.7%; non-target: 88.8%). Although the differences between target and non-target accuracies and interaction between two factors (target/non-target and number of averaging) were not significant (F = 0.9 and F = 1.3, respectively), the results suggest a possibility that averaging EEG signals is useful for detecting target direction.

**Table 5:** Across-subjects accuracy for target and non-target trials.

	Out-of-head sound localization				Loudspeaker			
	Target		Non-target		Target		Non-target	
Number of averaging	Mean [%]	SD	Mean [%]	SD	Mean [%]	SD	Mean [%]	SD
1	67.5	6.2	70.2	3.7	63.2	18.1	75.4	2.6
2	72.9	6.5	74.3	4.1	69.9	12.0	75.9	7.8
3	77.6	5.7	76.7	5.8	79.7	12.7	78.8	7.7
4	83.0	6.7	80.8	4.6	78.7	16.7	82.2	8.1
5	84.0	5.0	82.0	4.8	84.3	13.0	83.3	8.6
6	87.1	5.9	84.0	4.8	85.0	10.3	84.7	8.5
7	87.3	6.4	85.1	4.6	88.4	12.7	86.2	8.5
8	88.1	5.1	86.4	6.2	88.6	13.3	87.4	8.3
9	90.0	7.5	87.9	3.8	87.7	12.7	88.8	6.7
10	89.7	6.0	88.8	4.5	89.3	11.6	88.7	8.2

Classification accuracies for each direction in 10-trial averaged classifications are shown in Figure 6B. Mean accuracies across the subjects (black line) were almost the same, which showed more than 85%, although individual directional preference was observed. Similar results were confirmed when we used a different number for averaging. We performed a two-way repeated-measures ANOVA (factors: direction and number of averaging) and no difference and interaction between directions were found (F = 1.4 and F = 0.8, respectively) while the accuracy significantly increased along with number of averaging (F = 197.8, *p*<0.001). When we calculated the correlation coefficient between localization and classification accuracy across subjects and directions, no significance was observed (r = 0.26 for single-trial and 0.22 for 10-trial averaged classification). In contrast, the classification accuracies for each direction showed significant negative correlation with the counting error (*p*<0.05; r = −0.34 for single-trial and −0.32 for 10-trial averaging), suggesting a possibility that attentional states, i.e., how much the subject paid attention, but not localization accuracy are reflected by the counting and classification accuracy.

**Figure 7:**
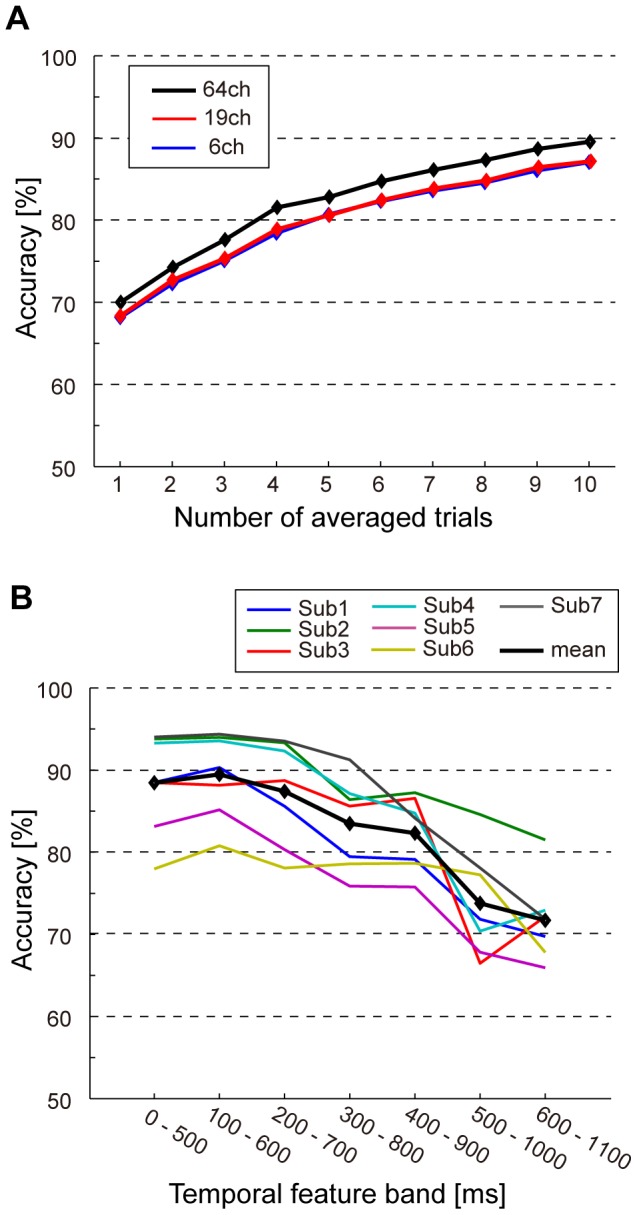
Accuracy when inputs for a classifier were changed. (A) Classification accuracy when the number of channels was reduced. The bold black, red, and blue lines indicate mean accuracy across subjects using 64 channels, 19 channels, and 6 channels, respectively. (B) Classification accuracy when different temporal features were used for classification. The data for 10-averaged trials is shown. Note that this analysis was done with 64 channels. Each colored line represents accuracy from an individual subject and the bold black line indicates the mean accuracy across subjects.

When we used EEG data for 19 out of 64 channels, the mean accuracy was similar to when we used 64 channels (Figure 7A). The difference between 64 and 19 channels (accuracy for 64 channels – for 19 channels) was −1.6% for the single-trial classification and 2.4% for 10-trial averaging (mean difference: −2.2%). Even when the EEG data for 6 channels were used, the classification accuracy for 6 channels did not decrease (mean difference: −2.4%). These results suggest that EEG signals in central and posterior areas are important for SVM classification.

In the classification analysis above, EEG data during 0 to 1100 ms were used as inputs (features). To see how the temporal information contributes to the classification, we examined the classification accuracy when we used a part of the temporal features in these EEG signals. In particular, based on the reasonable hypothesis that classification was achieved by the differences in the P300 responses, we used six different datasets, each of which had 500 ms samples and different data onset from 0 to 600 ms. Figure 7B showed classification accuracies in 10-trial averaging for each temporal feature band. Compared with a 0–1100 ms dataset, classification accuracy over 300–800 ms and 400–900 ms slightly deteriorated and those over 500–1000 ms and 600–1100 ms dramatically decreased while classification for the data from 0 to 200 ms remained the same as the accuracy using all the features (Figure 7B). Especially, the accuracy for a 100–600 ms dataset was almost same. This result suggests that temporal features around 200 ms and 500 ms, which probably reflects P300 responses and negative deflections (N2), have useful information for the classification.

### Comparison with the loudspeaker experiment

To examine whether auditory stimulus presentation using out-of-head sound localization is feasible, we also performed the same experiment using loudspeakers and compared the results.

In the localization test, the subjects recognized sound from all the directions accurately (Table 1 right). The correct rate was 99.7% and 7.3% higher than for the out-of-head sound localization experiment. Counting in the loudspeaker experiment also gave better results than out-of-head sound localization (Table 2 bottom). The error rate across subjects was just 2.2%.

The number of rejected trials was very small (Table 3 right). On average across the subjects 1.8±0.8% of the trials were excluded. We calculated the ERPs in the experiment with loudspeakers and compared them with those for the out-of-head sound localization. The averaged EEG waveform for target and non-target trials measured at electrode Pz was similar in both experiments (Figure S2), and we identified the differences between target and non-target trials. Latencies and amplitudes of the positive peak are shown in Table 4 (bottom). An ANOVA showed that, in the loudspeaker experiment, significantly larger amplitudes than for the out-of-head sound localization experiment were found (F = 23.8, *p*<0.005), while no difference in latency was observed (F = 2.7, *p* = 0.2).

**Figure 8:**
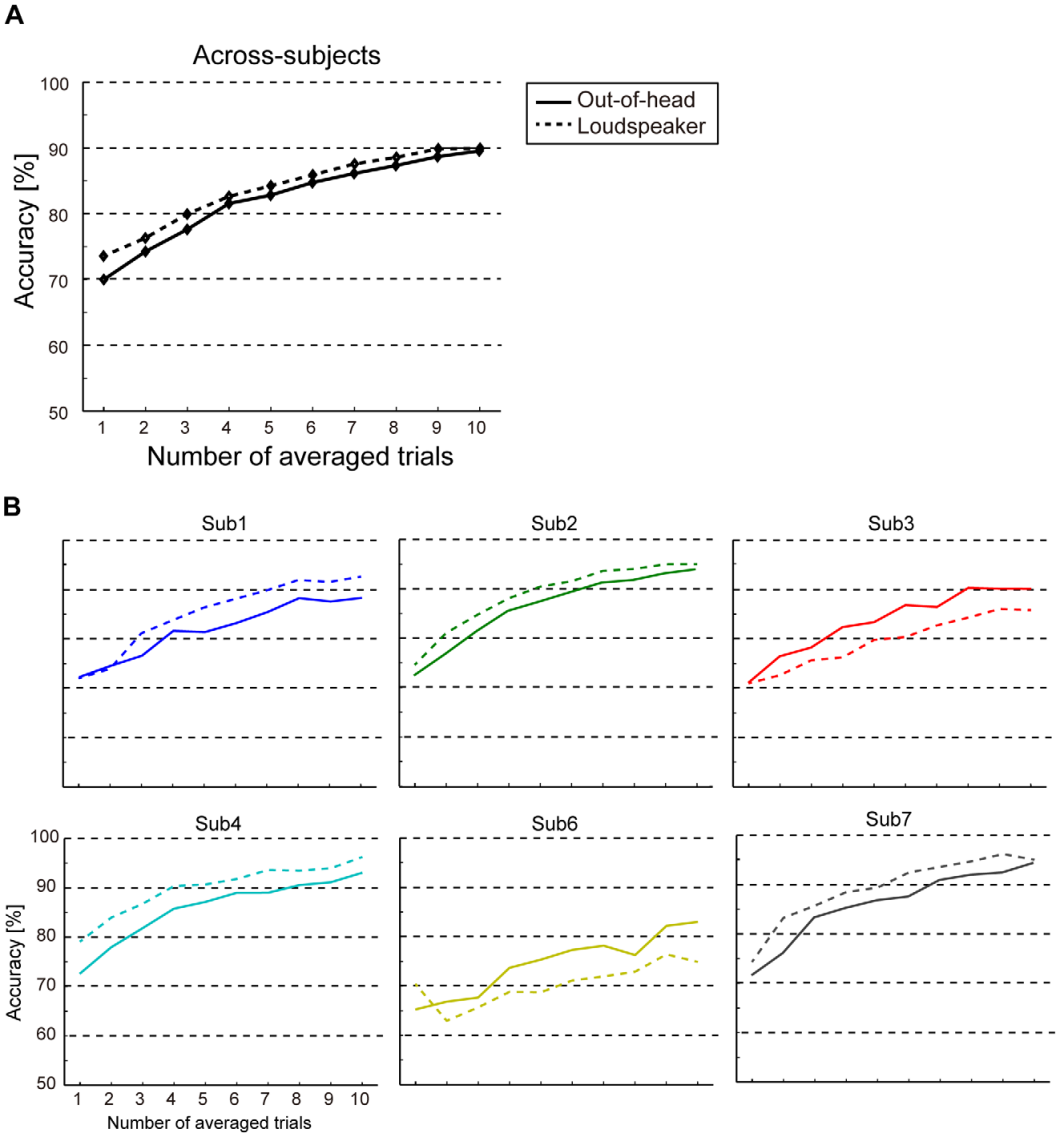
Comparison of accuracy between the out-of-head sound localization and loudspeaker experiments. (A) Mean classification accuracy across subjects. The solid black line shows accuracy obtained from the out-of-head sound localization experiment and the dashed black line indicates accuracy for the loudspeaker experiment. (B) Classification accuracy for each subject.

To examine how much the out-of-head sound localization techniques can be used for BCI compared with the loudspeaker experiment, we analyzed the same classification analysis for the EEG signals in the loudspeaker experiment. The mean classification accuracy across six subjects was almost the same as in the out-of-head sound localization experiment (Figure 8A). The accuracy in the out-of-head sound localization experiment was slightly higher than in the loudspeaker experiment. When we carefully looked at individual accuracies (Figure 8B), the performance in the out-of-head sound localization experiment was somewhat low (approximately 4%) for four subjects (Sub1, 2, 4, and 7) compared with the loudspeaker experiment. In contrast, the accuracy was slightly increased for two of the subjects (4.0% for Sub3 and 4.1% for Sub6 compared with the loudspeaker experiment across all the averaged-trials). A two-way ANOVA (stimulus presentation methods and number of averaging) revealed no significant difference between stimulus presentation methods (F = 0.2, *p* = 0.6) and no interaction (F = 1.2, *p* = 0.3), while the accuracy significantly increased with respect to the number of averaging (F = 102.3, *p*<0.001).

## Discussion

In the present study, we examined whether the attended direction for the subject can be predicted from EEG signals when auditory stimuli are presented using out-of-head sound localization. We found that the classification accuracy was 70.0% in a single-trial, and reached 89.5% when we used trial-averaged EEG signals. The results indicate that sound localization can be applied to a portable auditory BCI.

### Comparison with previous spatial auditory BCI

The experimental paradigm called spatial hearing was first proposed by Schreuder and his colleagues [11]. Comparing their study with ours, some similar results were obtained even when we used out-of-head sound localization. In their study, the auditory stimuli were presented from loudspeakers. Without EEG recording, they conducted a localization test for 8 directions spaced at 45° and the localization error was 7.4% on average. The localization errors in our study were 7.6% in the out-of-head sound localization experiment and 0.3% in the loudspeaker experiment across subjects and directions, suggesting that, similar to the previous study, the subjects could perceive auditory stimuli with greater than 90% accuracy. Because our experiment was conducted with 6 directions spaced at 60°, discrimination of the auditory stimuli was easier, resulting in higher localization with the loudspeaker experiment. Also, in terms of classification accuracy, our study obtained results similar to theirs, which showed approximately 60% in the single-classification and reached to 90% or more (in the C1000 condition; note that this is based on a rough comparison by visual inspection because they used a selection score but not classification accuracy, and the score is not shown). In their recent progress [12], they used spatial hearing with auditory stimuli from six directions as in our experimental setting, and examined the online performance of the BCI. They reported that the BCI performance for typing letters also reached more than 86% for the subjects who succeeded in all the sentences. Although there are many differences between their study and ours, for example online evaluation and the classifier, the levels of classification accuracy were similar.

Taken together with out-of-head sound localization and the loudspeaker experiment in the present study, spatial hearing with out-of-head sound localization is comparable to the loudspeaker setting and online application is also expected. Toward online application, the experimental paradigm should be sophisticated in improving localization accuracies or adopting shorter inter-stimulus-intervals, like a previous online study [12].

### Out-of-head sound localization and individual transfer function

To create a BCI using out-of-head sound localization requires users to accurately distinguish auditory stimuli from several different directions. It is generally known that spatial location of sound deviates from the actual location and sound localization becomes poor when the sound images are created using a mismatched transfer function. Therefore, to present the out-of-head sound image accurately, it is important to use a transfer function specifically fitted to each subject. For this reason, in this study, we measured the transfer function for each subject and used it to generate the auditory stimuli. As a result, the subject correctly reported the direction of sound in the localization test (Table 1), verifying that the out-of-head sound images were spatially localized. Compared with the results from the loudspeaker experiment, we found that the localization accuracies remained high in general although degradation in accuracy was observed. Furthermore, even in the out-of-head sound localization experiment, prominent P300 responses for the target trial were observed at the EEG electrodes, which were similar to those observed in the loudspeaker experiment. Therefore, these results demonstrated that the out-of-head sound localization using the individual's transfer function can be useful for an auditory BCI.

However, it is not easy to acquire the appropriate individual transfer function. One has to solve two issues to obtain it. First, the measurement of transfer functions requires a special acoustic environment. When we measured the individual transfer function, microphones were put in the canal and many loudspeakers were used to present auditory stimuli. In general, this environment and apparatus are not available. Second, the individual transfer function is not always fitted to the subject because of technical difficulty in measuring the transfer function. For example, sometimes earphones are not fitted to the subject, and this technical problem reduces the localization accuracy of the out-of-head sound images. In fact, one of the subjects (Sub6) in the present experiment seemed to have this problem, and this misalignment may have caused lower classification accuracy. Although the result of the localization test was not significantly poor for this subject, the accuracy was relatively low (85%, Table 1), and the number of targets counted was smaller than the number of targets actually presented (mean error across sessions: 18.9%, Table 2). The front-back confusion was also observed for this subject, which sometimes happens in the sound localization task using both virtual sound and loudspeakers (Figure 3B). Consistent with the behavioral data, ERP response was small and its latencies for some directions were large. Furthermore, the classification accuracy for Sub6 was not high: 65.2% in the single-trial classification and 82.9% in the 10-trial averaging. This was approximately 10% lower compared with the other subjects whose data achieved higher classification accuracy. From our results, we cannot conclude that these relatively poor performances for behavior and brain activity for Sub6 are attributed only to unfit transfer function because the other subjects showed good behavior performances but somewhat lower classification accuracy (Sub3) or vice versa (Sub2), but fitted individual transfer function may provide improvement of the BCI performances using out-of-head sound localization for some subjects.

An alternative way to overcome these problems is to use a standard library of transfer functions [27], [28], [29] and fit it to each subject. The standard library of transfer functions is a database for several subjects. If we use the standard library, we do not require a special acoustic environment. Because there is no guarantee that the standard library will fit a specific subject, we also need to develop a way of automatically improving the transfer function so that the out-of-head sound images can be localized accurately. Recently, such algorithms for learning the transfer function have been investigated [30] and may soon become available.

Thus, to accurately perceive virtual sound images, out-of-head sound localization is a successful technique, although it is necessary to measure it correctly, or find a way to fit a transfer function automatically using some algorithm.

### Classification in the out-of-head sound localization experiment

We examined whether EEG signals can predict the subject's attended direction for the presented out-of-head sound image, and found that classification accuracies using the out-of-head sound localization became high (Figure 6, Table 5). These performances were very similar to the accuracies reported in previous auditory BCI literature, which reported accuracies around 60 to 90% [8], although the experimental paradigm and conditions were different between the studies (e.g. the number of classes or iterations). To further compare performances in the present study with those obtained from previous literature, we evaluated the information transfer rate (ITR; bits per minute) [31]. Note that the ITR evaluated here is not completely the same because we used the bootstrap method to prepare trial-averaged EEG signals. In the single-trial classification, ITR became 9.2±1.1 (mean ± SD across subjects; 7.6 to 10.0 for each subject), and 1.7±0.2 for 10-trial averaging (1.4 to 1,9 for each subject). These ITRs were comparable to the high performance ITRs previously reported (ranging from 3 to 11 bits per minute) [11], [12], [13], [32]. Therefore, out-of-head sound localization will be useful for constructing a high-performance BCI system.

It is likely that these high performances can be achieved using the differences in the prominent P300 responses. We found that reducing channels from 64 to 19 or 6 did not greatly affect the classification accuracy. A previous study for feature selection reported that classification accuracy did not deteriorate even though only 6 channels were used [25], indicating the importance of the selected central and posterior channels for detecting P300 responses. Although the experimental paradigm and classification algorithm differ between their study and ours, we found similar results, i.e., classification performances from 6 and 19 channels were almost the same. In addition, when we reduced feature dimensions by choosing the time period of 100–600 ms for the classification analysis, the performances remained high. Since the signals during this period mostly reflected P300 responses during 200 to 500 ms, the result confirmed that EEG signals at the central and posterior part of the electrodes showed that P300 responses were evoked by the out-of-head sound images, especially for the representative 6 channels, and classification may be achieved mainly from these signals. The temporal feature band from 100–600 also includes negative ERP responses (probably N2 component). In agreement with previous auditory BCI [9], [11], [12], [13], [14], we found differences of these responses between average ERP responses for target and non-target trials (Figure 4). Therefore this early negative component may also contribute to the classification even when a stimulus was presented by out-of-head sound localization.

If we extract information related to these P300 and early negative component appropriately using some feature selection methods, the classification accuracy could be further improved. In the present study, a simple linear SVM was used for classification and any systematic feature selections were not considered because the feature selection was outside the scope of this study. Some studies showed that feature selection or spatial filtering improved classification accuracy [33], [34], [35], [36], [37]. We expect that applying feature selection will also improve classification accuracy in the out-of-head sound localization experiment.

### Advantages of auditory BCI using out-of-head sound localization

The auditory BCI using out-of-head sound localization has several advantages compared with the BCI using other types of characteristics. First, in agreement with previous studies using spatial hearing [11], [12], spatial information can be presented from any direction including the direction in which the user is not looking, i.e., it is also possible to perceive rearward directions. In fact, auditory stimuli were generated from directions behind the subject in our experiment, and the performances obtained were good. Needless to say, estimated spatial direction can be used not only for spelling devices but also for controlling a wheelchair. In the latter case, presenting auditory stimuli using out-of-head sound localization may be a valuable feature. Because this technique can present auditory cues for any direction in the horizontal plane, the user could control the wheelchair intuitively and easily by perceiving the direction in which they intend to go. Furthermore, 3D spatial auditory cues can theoretically be presented using out-of-head sound localization. Presenting 3D auditory stimuli is apparently difficult using loudspeaker setting. Thus, using out-of-head sound localization enables the BCI to increase the number of classes. This may lead to improved resolution (ITRs) since ITRs become higher in general with an increasing number of classes. In addition to this, out-of-head sound localization allows the user to use the BCI anywhere. It has been thought that an auditory BCI using spatial hearing with loudspeakers is useful for end-users at the bedside but not suitable for portable users because of the equipment setting required [12]. However, if we incorporate out-of-head sound localization into the spatial auditory BCI, it may be possible to create a portable high-performance BCI, which inherits the advantages of a loudspeaker setting. Therefore, out-of-head sound localization may extend the spatial hearing paradigm and provide a portable auditory BCI.

By combining the spatially presented out-of-head sound images with other characteristics of auditory stimuli, the auditory BCI will become more powerful. Previous auditory BCIs used pitch, amplitude, or different types of voice, and some studies combined spatial information with these characteristics as a cue. For example, a recent pioneering BCI study generated auditory stimuli with a different pitch from different directions (left or right), and significantly increased the number of classes that could be classified [13]. Although we presented white noise stimuli in this study, it is possible to generate other types of tone or natural stimuli. If we use them, it will promote fine control of external devices and quicker and more accurate communication. Likewise, auditory streaming is a useful approach for clinical and daily use [9], [10], which could be combined with out-of-head sound localization. Thus, further investigation is needed for choosing optimal stimulus selection and combination, or developing an experimental design such as shortening the inter-stimulus interval to improve accuracy. Online classification is also necessary for the evaluation and development of a BCI using out-of-head sound localization.

## Conclusion

We investigated the possibility of an auditory BCI using the out-of-head sound localization technique. Using EEG signals from 64 electrodes, we were able to classify whether the subject directed his attention toward the direction of sound or not. In the single-trial classification, mean accuracy across subjects became 70.0% and its ITR was 9.2. When we used averaged signals as inputs to the classifier, the mean accuracy across seven subjects reached 89.5% (for 10-trial averaging). Further analysis revealed that P300 and early negative responses measured at the central and posterior part of the electrodes contributed to the classification. These classification performances were comparable to those obtained from the loudspeaker experiment. Thus, we demonstrated that a high-performance and loudspeaker-less P300-BCI system can be achieved using out-of-head sound localization. We expect that the performance will be improved by developing the design of the auditory stimuli and experimental setting. Because this study performed only offline analysis, online classification is also required in the development of future systems.

## Supporting Information

Text S1 **Measurement of transfer functions.** Detailed measurement environment and processing to obtain transfer functions are described.
(DOC)

Figure S1 **Principles of out-of-head sound localization and an environment of transfer function measurement.** (A) Sound field with loudspeakers. (B) Simulation through earphones. (C) Measurement environment in the test room.
(TIF)

Figure S2 **Averaged ERPs at Pz in the loudspeaker experiment.** Each figure shows the averaged ERP responses at electrode Pz for the loudspeaker experiment. The accuracy for each subject is shown in each column (Sub1–Sub7 except for Sub5). The red line shows the ERP for target trials and the black line shows the ERP for non-target trials.
(TIF)
