# Supplementary material for: Estimating the Intended Sound Direction of the User: Toward an Auditory Brain-Computer Interface Using Out-of-Head Sound Localization
Source: PLoS One. 2013 Feb 20;8(2):e57174. doi: 10.1371/journal.pone.0057174 (PMC3577758; doi:10.1371/journal.pone.0057174)
Supplement: Text S1 — Measurement of transfer functions. Detailed measurement environment and processing to obtain transfer functions are described. (DOC) [file pone.0057174.s001.doc]

**Estimating the Intended Sound Direction of the User: toward an Auditory Brain-Computer Interfaces Using Out-of-head Sound Localization**

**Supporting Text S1: Measurement of transfer functions**

The principle of out-of-head sound localization is to reproduce the sound waveforms of the actual sound field at the listener’s eardrums using stereo earphones or headphones. To achieve out-of-sound localization, we need to 1) measure the impulse responses of the Spatial Sound Transfer Functions (SSTFs) and Ear Canal Transfer Functions (ECTFs), 2) obtain the Sound Localization Transfer Functions (SLTFs), and 3) produce the out-of-head sound using the SLTFs. Each transfer function was modeled for each ear.

First, we measured impulse responses of subject-specific SSTFs and ECTFs. The SSTF models the conversion of the input signals of the loudspeaker into the output signals of the microphone in the ear canal of the subject (Figure S1A), and ECTF (Ear Canal Transfer Function) models the conversion of the input signals of the earphone into the output signals of the microphone in the ear canal of the subject (Figure S1B). Note that here we defined SLTFs as Head Related Transfer Functions (HRTFs) since the definition of the SLTF is similar to that of the HRTF.

Measurement of the SSTF for the time domain was performed in the test room at the Nagaoka National College of Technology (Figure S1C). The reverberation time in the test room was about 0.1 s. Twenty-four loudspeakers (MODEL SD-0.6, EMIC, Japan) were placed at 15° intervals, and the distance from the center of the subject’s head to the face of the each loudspeaker was 1.5 m. A small microphone (UC-92, RION, Tokyo, Japan) was placed at the entrance to each ear canal. Two matched microphones were used, and responses from both were measured simultaneously at 44.1 kHz. To measure the SSTFs and ECTFs, we used pseudo white noise generated by the Maximum length sequence method (M-sequence; order: 16; number of samples: 65535; frequency range: 100 Hz–18 kHz). The subject sat on a seat and pseudo white noise was radiated from the loudspeakers through an amplifier (1200VI, BOSE, Framingham, MA, USA). The sound pressure level was adjusted to 65 dB. To reduce the measurement noise, a grand average was calculated using three or four trials of the measured responses.

In the measurement of the ECTFs for the time domain, the subject wore earphones (MDR-ED238, Sony, Tokyo, Japan) and sound (pseudo white noise) was presented through the earphones. The procedures for the measurement were the same as those for SSTFs.

Impulse responses of SSTFs and ECTFs, i.e. SSIR (Spatial Sound Impulse Responses) and ECIR (Ear Canal Impulse Response) were then calculated using the following three steps: Adamal Conversion, removal of DC components, and correction of distortion by sample-and-hold. The SSIR and ECIR were transformed into SSTF and ECTF for the frequency domain.

Next, the SLTF, a function from input signals (sounds) to each ear canal (Figure S1B), for the frequency domain was defined according to the following relationship:

$$SLTF = SSTF\cdot ECTF^{-1}.$$

The frequency range of the SLTF was confined to 100 Hz to 15 kHz. An impulse response of SLTF (i.e. Sound Localized Impulse Response: SLIR) was then calculated for the time domain.

Finally, the SLIR (first 2048 samples) was convolved with the auditory stimuli that were presented to the subject (white noise in the present experiment). Thus, the out-of-head sound image was created.
