# Supplementary figures and images for: Estimating the Intended Sound Direction of the User: Toward an Auditory Brain-Computer Interface Using Out-of-Head Sound Localization
Source: PLoS One. 2013 Feb 20;8(2):e57174. doi: 10.1371/journal.pone.0057174 (PMC3577758; doi:10.1371/journal.pone.0057174)

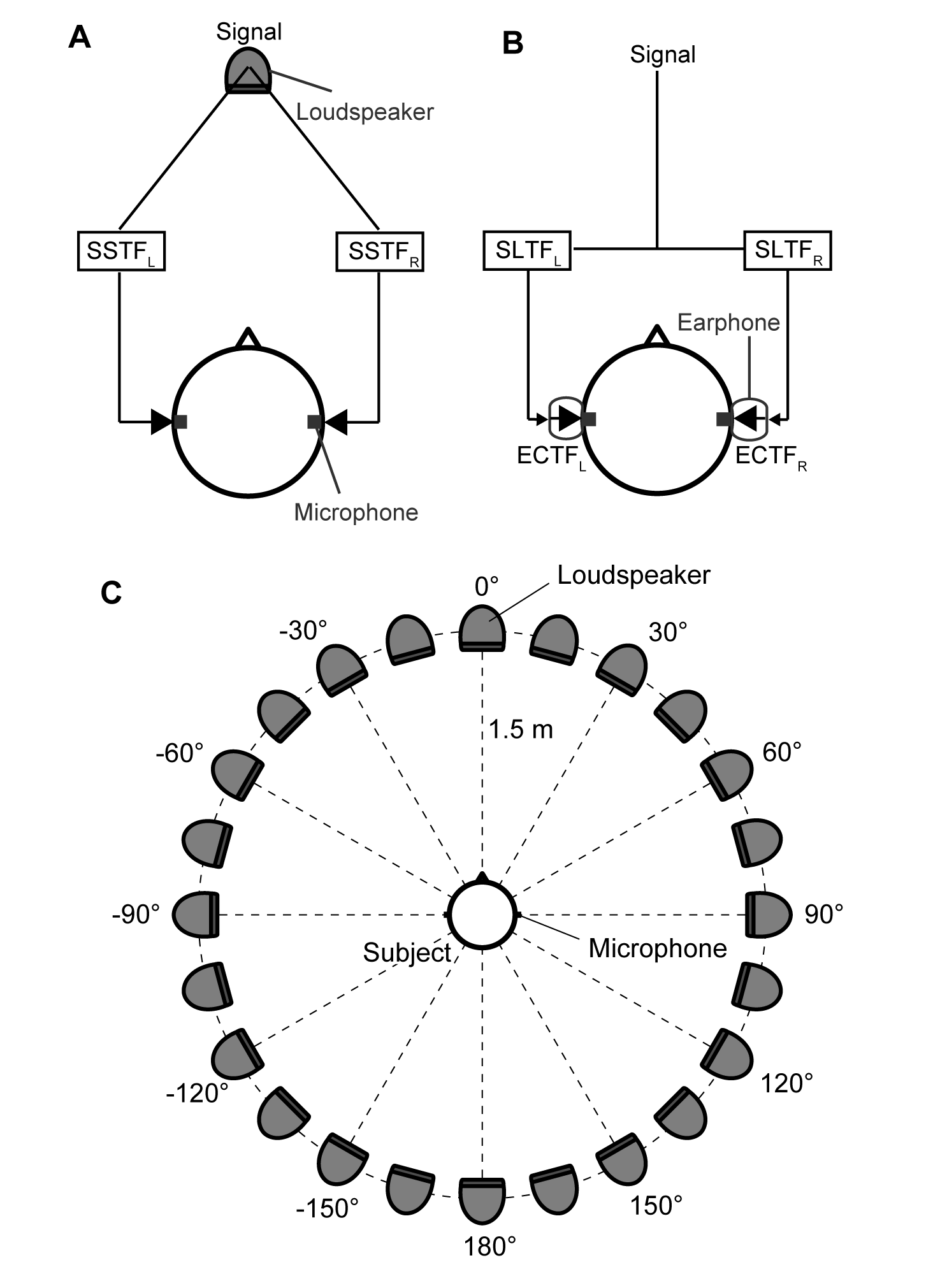

Supplement: Figure S1 — Principles of out-of-head sound localization and an environment of transfer function measurement. (A) Sound field with loudspeakers. (B) Simulation through earphones. (C) Measurement environment in the test room. (TIF) [file pone.0057174.s002.tif]

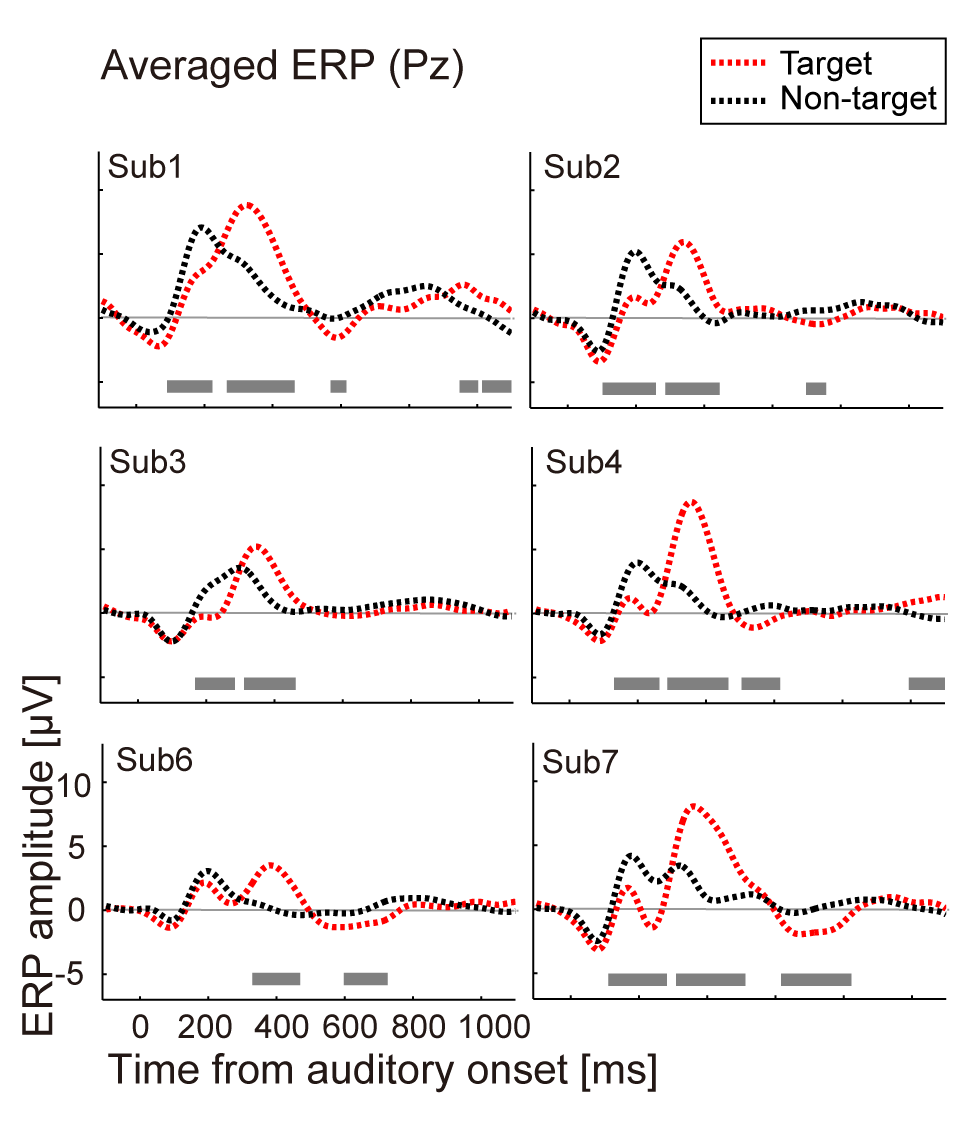

Supplement: Figure S2 — Averaged ERPs at Pz in the loudspeaker experiment. Each figure shows the averaged ERP responses at electrode Pz for the loudspeaker experiment. The accuracy for each subject is shown in each column (Sub1–Sub7 except for Sub5). The red line shows the ERP for target trials and the black line shows the ERP for non-target trials. (TIF) [file pone.0057174.s003.tif]
